# Chronic growth faltering amongst a birth cohort of Indian children begins prior to weaning and is highly prevalent at three years of age

**DOI:** 10.1186/1475-2891-8-44

**Published:** 2009-09-29

**Authors:** Andrea M Rehman, Beryl P Gladstone, Valsan P Verghese, Jayaprakash Muliyil, Shabbar Jaffar, Gagandeep Kang

**Affiliations:** 1 MRC Tropical Epidemiology Group, London School of Hygiene & Tropical Medicine, London, WC1E 7HT, UK; 2 Department of Community Health, Christian Medical College, Vellore, 632 004, India; 3 Department of Child Health, Christian Medical College, Vellore, 632 004, India; 4 Department of Gastrointestinal Sciences, Christian Medical College, Vellore, 632 004, India

## Abstract

**Background:**

Poor growth of children in developing countries is a major public health problem associated with mortality, morbidity and developmental delay. We describe growth up to three years of age and investigate factors related to stunting (low height-for-age) at three years of age in a birth cohort from an urban slum.

**Methods:**

452 children born between March 2002 and August 2003 were followed until their third birthday in three neighbouring slums in Vellore, South India. Field workers visited homes to collect details of morbidity twice a week. Height and weight were measured monthly from one month of age in a study-run clinic. For analysis, standardised z-scores were generated using the 2006 WHO child growth standards. Risk factors for stunting at three years of age were analysed in logistic regression models. A sensitivity analysis was conducted to examine the effect of missing values.

**Results:**

At age three years, of 186 boys and 187 girls still under follow-up, 109 (66%, 95% Confidence interval 58-73%) boys and 93 (56%, 95% CI 49-64%) girls were stunted, 14 (8%, 95% CI 4-13%) boys and 12 (7%, 95% CI 3-11%) girls were wasted (low weight-for-height) and 72 (43%, 95% CI 36-51) boys and 66 (39%, 95% CI 31-47%) girls were underweight (low weight-for-age). In total 224/331 (68%) children at three years had at least one growth deficiency (were stunted and/or underweight and/or wasted); even as early as one month of age 186/377 (49%) children had at least one growth deficiency. Factors associated with stunting at three years were birth weight less than 2.5 kg (OR 3.63, 95% CI 1.36-9.70) 'beedi-making' (manual production of cigarettes for a daily wage) in the household (OR 1.74, 95% CI 1.05-2.86), maternal height less than 150 cm (OR 2.02, 95% CI 1.12-3.62), being stunted, wasted or underweight at six months of age (OR 1.75, 95% CI 1.05-2.93) and having at least one older sibling (OR 2.00, 95% CI 1.14-3.51).

**Conclusion:**

A high proportion of urban slum dwelling children had poor growth throughout the first three years of life. Interventions are needed urgently during pregnancy, early breastfeeding and weaning in this population.

## Background

In developing countries, poor growth of children under five is a major public health problem. Children with poor growth have high rates of mortality and morbidity [1,2] and can suffer motor and mental developmental delay [3,4]. One of the WHO's millennium development goals is to halve by 2015 the proportion of children under five who are stunted, wasted or underweight from 1990 levels. In 2005, the WHO estimated that of all children under five living in developing countries, 32% were stunted and 10% were wasted [5] using the WHO's new child growth standards [6]. In a nationwide survey in 2005-6, Indian estimates of stunting were 38%, of wasting were 19% and of underweight were 46% among children under 3 years of age [7]. A different growth standard to the WHO was used to calculate the Indian estimates [8], which may have underestimated the proportion of children with poor growth [9,10]. India therefore has a larger than average proportion of children who are stunted, wasted and underweight.

There are large urban slums in India and it has been suggested that between 50-94% of children are underweight in different slum populations in Northern India [11]. However the studies which have reported these findings were mostly cross-sectional, and were varied in the growth standards used, so have limited use when comparing with other estimates.

We have conducted a longitudinal birth cohort in an urban slum community in Vellore, South India and measured children's growth monthly for three years. We describe here growth throughout the first three years of life and the factors related to poor growth within this population.

## Methods

The design of the study has been reported previously [12,13]. In brief, the primary aim of the study was to investigate the natural history of rotavirus infections in children. The study was conducted in three neighbouring urban slums in Vellore measuring 2.2 sq. km with a population density of approximately 17,000 per sq. km. A common occupation in the area is 'beedi work', the manual production of cigarettes for a daily wage. Pregnant women were identified during a survey conducted in 2002. Infants were recruited from birth between March 2002 and August 2003 following written informed consent from the mother. Those living in brick-built houses with more than four rooms (n = 46) or who had birth weight less than 1.5 kg (n = 2) were excluded. Socio-economic status within this relatively homogenous population was stratified as previously described [13]. Field workers visited infants at home soon after birth and twice weekly thereafter collecting details of morbidity and referring children to a physician-run study clinic as necessary. Follow-up continued until the child's third birthday. The last child was followed up in August 2006. Breastfeeding uptake was high in this population with 445 (98%) of the 452 enrolled children known to start.

Height and weight at birth, where available, were obtained from delivery records available at the first home visit. Subsequently, height and weight were obtained by field workers at the study clinic using single measurements. Weight was measured using a Salter weighing scale to the nearest 100 grams. Recumbent length was measured using a standard infantometer up to the child's first birthday or until the child was able to stand, and subsequently using a stadiometer, both to the nearest millimetre. The instruments were calibrated at least once a week. Field workers were retrained and procedures standardised once every three months.

We calculated total duration of illness; we defined major illness as diarrhoea, lower respiratory infection, tuberculosis, jaundice, central nervous system infection, seizures or convulsions, neonatal sepsis, neonatal jaundice, congenital diseases, burns, scalds, fracture, crush injuries, dengue, and physician diagnosed malnutrition; and defined minor illness as fever, cough or cold, asthma, wheezing, bronchiolitis, exanthematous fever (except measles), skin morbidities, eye morbidities, ear, nose and throat morbidities, incessant crying, abrasions and bites, pica, aphthous ulcer, localised infections, anaemia and loss of appetite. These comprised caregiver diagnosis of minor illness during field worker visits and physician diagnosis of minor and major illness from visits to the study clinic and from hospital admissions [13].

Respiratory illness was common, occurring at a rate of 7.4 episodes per child year (and a median of 48 days of illness) in the first year of life [13], 7.1 episodes (and a median of 67.5 days of illness) in the second year of life and 6.6 episodes (and a median of 50 days of illness) in the third year of life (Gladstone *et al*. unpublished). Gastro-intestinal illness was more common in the first year of life than in the second and third years, occurring at a rate of 3.6 episodes per child year (and a median of 8 days of illness) in the first year of life [13], 1.6 episodes (and a median of 3 days of illness) in the second year of life and 1.2 episodes (and a median of 1 day of illness) in the third year of life (Gladstone *et al*. unpublished). Peak rates of illness were experienced between 3 and 5 months of age, around the time of weaning [13]. The rate of hospitalisation was low, 0.28 per child year in the first year, 0.11 in the second year and 0.07 in the third (Gladstone *et al*. unpublished).

Poor growth was quantified as the percentage of children stunted (low height-for-age), wasted (low weight-for-height) and underweight (low weight-for-age). 'Low' was characterised as less than -2 standard deviations from the growth reference. We used the 2006 WHO child growth standards [6] as the reference population to calculate height-for-age (HAZ), weight-for-height (WHZ) and weight-for-age (WAZ) z-scores. Plots of individual's growth curves revealed some discrepancies in height measurements. Therefore measurements which were more than three standard deviations different from the nearest four measurements were set to missing height *a priori*. All measurements relate to attained month of age, for example a measurement taken on a day of life between six times 30.4375 (183 days) and seven times 30.4375 (213 days) relates to six months of age. The first measurement in an attained month of age was used in analysis.

As stunting is considered to be a long-term deficit in growth and it was the dominant growth faltering seen at three years of age, we investigated the probability of being stunted at three years using logistic regression with potential risk factors defined *a priori*. We investigated whether temporal changes in both duration of major illness or duration of all illness were associated with stunting at 36 months, but found no patterns. We therefore present the association with total duration of major illness over three years. We investigated causes of missing values, in both stunting at 36 months and in potential risk factors, using observed data. To be conservative we included all potential risk factors in a multivariate model, whether associated with the outcome or with missing values [14]. We investigated whether risk factors were gender-dependent by testing interaction terms between the risk factor and gender including interactions with likelihood ratio p < 0.05. We conducted both a complete case analysis (including only children who had no missing values in the outcome or potential risk factors) and for comparison an analysis where all missing values were imputed using chained equations [15-17] [see additional file 1]. We then carried out a sensitivity analysis to establish the stability of the estimated odds ratios [see additional file 2]. Finally, we also investigated risk factors for early growth faltering (stunted, wasted or underweight) at six months of age using the same methodology and investigating the relationship between growth faltering and all illness in the first six months of life [see additional file 3]. All analyses were undertaken using STATA version 10.1 [18]. The study was approved by the Institutional Review Boards of the London School of Hygiene and Tropical Medicine, London, UK, Baylor College of Medicine, Houston, USA, and Christian Medical College, Vellore, India.

## Results

There were 914 pregnancies eligible and 48 ineligible for the study. A total of 462 (48%) mothers refused, primarily due to the common practice of pregnant women returning to their mother's house to give birth and stay for three months afterwards, thus making their children not available for follow up according to the study protocol. Mothers who were not recruited to the study did not differ from mothers who were recruited in terms of their age, socio-economic status and their child's birth weight and gender.

Between March 2002 and November 2003 452 children began follow-up. By six months of age 403 (89%) were still under follow-up and 373 (83%) were still under follow-up at three years of age. Of the 79 children that left the study, 44 moved away from the study area and 30 discontinued mainly because of the fear of serum collection. There were five deaths, including three children who suffered diarrhoea and dehydration in their first year of life [13] and two children who died in hospital, one of a congenital cardiopulmonary disorder in their first year of life and one of seizures in their third year of life. The median (Inter-quartile range: IQR) age on leaving the study was 4.1 months (2.5-11.2 months). Children who did not complete follow-up had less educated mothers (chi-square p = 0.064) and started complementary foods later (log-rank test p = 0.0004) than children who completed follow-up.

There were 14464 scheduled anthropometry measurements. Height measurements used for analysis were measured on 12705 (88%) occasions and weight measurements used for analysis were measured on 13083 (90%) occasions. Fifty four (0.004%) height measurements were set to missing due to discrepancy and 1461 (10%) measurements taken later in the same attained month of age as another measurement were excluded from analysis as described above.

Two hundred and 81 (62%) mothers were of low socio-economic class with the remainder of lower middle class. Mean age of the mothers was 24 (SD: 4) years. Most (313, 69%) mothers had received at least some primary education. The mean height of 363 mothers who had height available was 152 cm (SD: 6.6 cm). The Kaplan-Meier estimate of the median age complementary foods were introduced was 3.0 months (IQR: 1.9-4.0 months).

**Figure 1:**
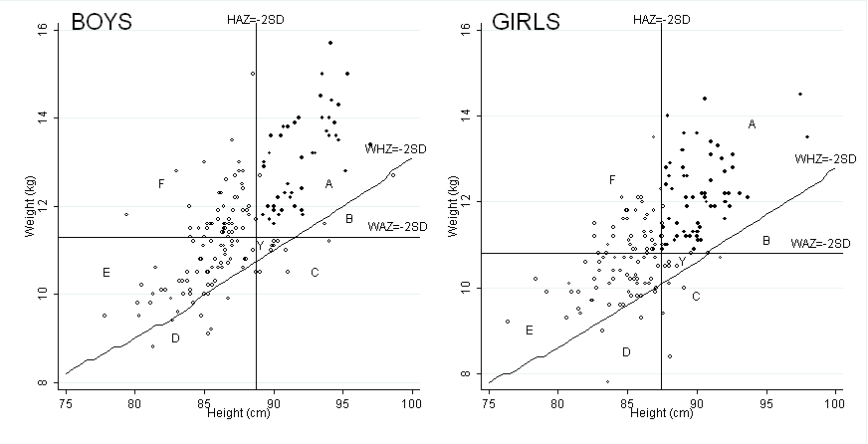
**Distribution of growth faltering at 36 months of age according to WHO growth standards**. Key- A: Not malnourished, B: Wasted only, C: Wasted and underweight, D: Wasted, stunted and underweight, E: Stunted and underweight, F: Stunted only, Y: Underweight only. Open circles denote children with at least one growth faltering, closed circles denote children with normal growth.

Table 1, Table 2, and Figure 1 show the growth data for boys and girls. By age 36 months, 109 (66%) boys and 93 (56%) girls were stunted, 14 (8%) boys and 12 (7%) girls were wasted and 72 (43%) boys and 66 (39%) girls were underweight. By 36 months of age, 120 boys, 72% (95% CI: 65-79%), and 105 girls, 63% (95% CI: 56-71%), were either stunted or underweight or wasted, providing the overall burden of malnutrition, according to the 2006 WHO standards. The percentages stunted from above can be broken down into three categories, 46 (28%) boys and 38 (23%) girls were stunted only (F), 55 (33%) boys and 48 (29%) girls were stunted and underweight (E) and 8 (5%) boys and 7 (4%) girls were stunted, underweight and wasted (D). There are four other growth categories shown in Figure 1, children who were not stunted, wasted or underweight, 46 (28%) boys and 62 (37%) girls (A), children with only wasting, 2 (1%) boys and 0 (0%) girls (B), children who were wasted and underweight, 4 (2%) boys and 5 (3%) girls (C), and children who were only underweight, 5 (3%) boys and 6 (4%) girls (Y).

**Table 1:** The number of boys and girls studied and growth from birth to 12 months of age

**Boys**	**Attained age in months**			
	**0 (Birth)**	**3**	**6**	**12**
*Number followed*	227	209	202	198
*Number of measurements*				
Weight	223	185	187	166
Height	55	181	186	165
*Mean (SD)*				
Weight (kgs)	3.0 (0.4)	5.7 (0.9)	7.0 (1.0)	8.3 (1.1)
Length/height (cms)	47.6 (3.1)	59.7 (3.6)	64.9 (3.2)	70.8 (3.4)
Weight gain^1 ^(kgs)		2.7 (0.8)	1.5 (0.9)	1.4 (0.7)
% Weight gain^1^		94.4 (29.2)	33.4 (34)	20.3 (12.7)
Length/height gain^1 ^(cms)		11.8 (3.3)	5.2 (3.2)	6.0 (2.7)
% Length/height gain^1^		24.8 (7.4)	9.0 (6.2)	9.3 (4.6)
Height-for-age z-score	-1.2 (1.7)	-1.5 (1.7)	-1.6 (1.5)	-2.0 (1.4)
Weight-for-height z-score	-0.4 (1.7)	-0.5 (1.7)	-0.5 (1.6)	-0.7 (1.5)
Weight-for-age z-score	-0.9 (0.9)	-1.5 (1.3)	-1.4 (1.3)	-1.5 (1.2)
*Percent (95% CI) growth faltering*				
Stunted (HAZ<-2)	28 (15-41)	35 (28-43)	37 (30-44)	50 (42-58)
Wasted (WHZ<-2)	12 (2-22)	18 (12-24)	16 (10-21)	16 (10-22)
Underweight (WAZ<-2)	12 (7-16)	33 (26-40)	34 (27-41)	34 (26-41)
**Girls**				
*Number followed*	225	212	201	193
*Number of measurements*				
Weight	218	182	185	179
Length/Height	54	177	184	179
*Mean (SD)*				
Weight (kgs)	2.9 (0.4)	5.2 (0.7)	6.4 (0.8)	7.7 (0.9)
Length/height (cms)	47.8 (2.1)	58.8 (2.9)	63.4 (2.9)	69.7 (3.1)
Weight gain^1 ^(kgs)		2.4 (0.6)	1.4 (0.8)	1.4 (0.5)
% Weight gain^1^		84.9 (27.3)	32.2 (32.9)	21.6 (9.3)
Length/height gain^1 ^(cms)		11.6 (2.8)	5.1 (3.3)	6.2 (2.6)
% Length/height gain^1^		24.5 (6.3)	9.0 (6.7)	9.9 (4.5)
Height-for-age z-score	-0.7 (1.1)	-1.1 (1.3)	-1.3 (1.3)	-1.6 (1.2)
Weight-for-height z-score	-0.6 (1.3)	-0.7 (1.5)	-0.6 (1.4)	-0.7 (1.2)
Weight-for-age z-score	-0.8 (1.0)	-1.4 (1.0)	-1.4 (1.0)	-1.4 (1.0)
*Percent (95% CI) growth faltering*				
Stunted (HAZ<-2)	9 (1-18)	22 (16-28)	26 (19-32)	39 (32-47)
Wasted (WHZ<-2)	12 (2-22)	17 (11-22)	10 (5-14)	15 (9-20)
Underweight (WAZ<-2)	11 (6-15)	27 (20-34)	25 (19-32)	26 (19-32)

^1^Gain from previous attained age in the table, for example under 6 months of age the gain is between 3 and 6 months of age

**Table 2:** The number of boys and girls studied and growth from 18 to 36 months of age

**Boys**	**Attained age in months**			
	**18**	**24**	**30**	**36**
*Number followed*	191	191	187	186
*Number of measurements*				
Weight	174	162	168	166
Height	174	162	168	166
*Mean (SD)*				
Weight (kgs)	9.2 (1.2)	10.0 (1.2)	10.9 (1.2)	11.7 (1.3)
Length/height (cms)	75.6 (3.1)	79.7 (3.3)	83.7 (3.5)	87.7 (3.7)
Weight gain^1 ^(kgs)	0.9 (0.6)	0.8 (0.5)	0.8 (0.5)	0.8 (0.4)
% Weight gain^1^	10.6 (6.7)	9.1 (5.2)	8.1 (4.8)	7.4 (4.2)
Length/height gain^1 ^(cms)	4.8 (2.3)	4.1 (2.4)	4.0 (1.6)	4.0 (1.6)
% Length/height gain^1^	6.9 (3.5)	5.4 (3.1)	5.0 (2.1)	4.8 (1.9)
Height-for-age z-score	-2.3 (1.1)	-2.5 (1.1)	-2.5 (1.0)	-2.3 (1.0)
Weight-for-height z-score	-0.8 (1.2)	-0.7 (1.1)	-0.7 (1.0)	-0.8 (1.0)
Weight-for-age z-score	-1.7 (1.1)	-1.8 (1.0)	-1.8 (1.0)	-1.8 (0.9)
*Percent (95% CI) growth faltering*				
Stunted (HAZ<-2)	68 (61-76)	69 (62-77)	71 (64-79)	66 (58-73)
Wasted (WHZ<-2)	14 (8-19)	8 (4-13)	7 (3-11)	8 (4-13)
Underweight (WAZ<-2)	44 (37-52)	46 (38-54)	47 (39-55)	43 (36-51)
**Girls**				
*Number followed*	190	189	188	187
*Number of measurements*				
Weight	176	168	168	166
Length/Height	176	168	168	165
*Mean (SD)*				
Weight (kgs)	8.7 (1.0)	9.5 (1.1)	10.4 (1.2)	11.2 (1.2)
Length/height (cms)	74.3 (3.1)	78.6 (2.8)	83.2 (3.2)	87.1 (3.4)
Weight gain^1 ^(kgs)	0.9 (0.4)	0.9 (0.5)	0.8 (0.5)	0.8 (0.5)
% Weight gain^1^	12.1 (5.9)	10.5 (6.2)	8.7 (5.0)	7.6 (4.4)
Length/height gain^1 ^(cms)	4.5 (2.3)	4.2 (2.2)	4.5 (1.9)	3.9 (1.7)
% Length/height gain^1^	6.5 (3.6)	5.7 (3.1)	5.7 (2.5)	4.7 (2.1)
Height-for-age z-score	-2.1 (1.1)	-2.3 (0.9)	-2.2 (0.9)	-2.1 (0.9)
Weight-for-height z-score	-0.7 (1.0)	-0.6 (0.9)	-0.7 (0.9)	-0.8 (0.9)
Weight-for-age z-score	-1.5 (0.9)	-1.7 (0.9)	-1.8 (1.0)	-1.8 (0.9)
*Percent (95% CI) growth faltering*				
Stunted (HAZ<-2)	54 (46-61)	61 (53-68)	60 (52-68)	56 (49-64)
Wasted (WHZ<-2)	8 (4-12)	4 (1-8)	7 (3-11)	7 (3-11)
Underweight (WAZ<-2)	31 (24-38)	37 (29-45)	43 (35-51)	39 (31-47)

^1^Gain from previous attained age in the table, for example under 24 months of age the gain is between 24 and 18 months of age; under 18 months of age the gain is from the last age in Table 1, from 12 months

**Figure 2:**
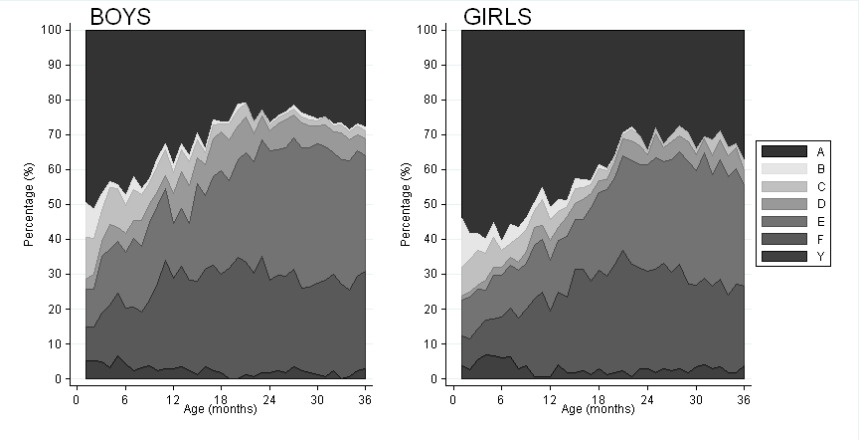
**Distribution of growth faltering categories over the first three years of life**. Key- A: Not malnourished, B: Wasted only, C: Wasted and underweight, D: Wasted, stunted and underweight, E: Stunted and underweight, F: Stunted only, Y: Underweight only.

Of children with both height and weight measured and still under follow-up, there were 186/377 (49%) aged one month who had at least one growth faltering (stunting, wasting or underweight) (Figure 2). By 20 months of age, this percentage had increased steadily with 253/355 (71%) of children faltering. From 20 months to 36 months of age the percentage of children faltering remained relatively stable so that by 36 months of age 224/331 (68%) had at least one growth faltering. The percentages of children who were stunted, wasted and underweight changed throughout the 36 month period with proportionately more wasted and underweight children in the first year of life compared to more stunted children in the second and third years of life.

We also investigated all month-to-month changes in growth faltering and recovery states by examining the probabilities of transitioning among the seven categories described above (Table 3). The majority of children with normal growth continued to grow normally in the following month (of the month-to-month transitions, 3519/4127 (85%)). Similarly, the majority of children who were stunted only, stunted and underweight only, or stunted, wasted and underweight remained in the same state in the following month.

**Table 3:** Month-to-month transition probabilities^1 ^between the growth categories including all months of age from 1^2 ^to 36 months by gender, A: Not malnourished, B: Wasted only, C: Wasted and underweight, D: Wasted, stunted and underweight, E: Stunted and underweight, F: Stunted only, Y: Underweight only

**Boys**			**Growth faltering category**						
	**A**	**B**	**C**	**D**	**E**	**F**	**Y**	**% Total**	**Total occurrences**
A	85	1.4	1.6	0.1	1.4	9	1.8	100	1790
B	31	52	15	0	0	0	2.1	100	97
C	10	1.4	62	10	5	1.0	10	100	292
D	0.5	0	1.9	75	23	0	0.3	100	374
E	1.4	0	1.5	5	82	8	1.2	100	1452
F	11	0.1	0.1	0.2	9	79	0.7	100	1333
Y	18	0.8	16	1.5	25	5	34	100	133
% Total	32	1.5	5	7	27	25	2.5	100	5471

**Girls**									
	**A**	**B**	**C**	**D**	**E**	**F**	**Y**	**% Total**	**Total occurrences**

A	86	1.6	1.2	0.04	1.0	9	1.7	100	2337
B	43	41	13	0	0	0	2.4	100	127
C	13	1.8	56	8	8	0.9	13	100	228
D	0.5	0	2.3	73	24	0	0.5	100	222
E	1.9	0.2	2.1	4	81	8	2.3	100	1221
F	14	0.2	0.3	0	9	77	0.5	100	1281
Y	23	0.7	7	1.3	34	6	29	100	155
% Total	42	1.8	4	4	22	23	2.7	100	5571

^1^Of any two consecutive months from 1 month to 36 months, growth category in the first of those months along the left hand side, growth category transitioned to in the following month along the top. For example of the 1790 occurrences of normal growth among boys (group A on the left hand side), 85 times in 100 the child would stay with normal growth in the following month (group A along the top), and 9 times in 100 the child moved to be stunted only in the following month (group F along the top). Of the 1333 occurrences of stunting only among boys (group F on the left hand side), 79 times in 100 the child remained stunted only the following month (group F along the top) and 11 times in 100 the child moved to have normal growth in the following month (group A along the top).

^2 ^Birth was not included due to the high number of missing values for length

In the crude (Table 4) and multivariate models (Table 5) investigating risk factors for stunting at 36 months, we found five factors (low birth weight, beedi work in the household, maternal height less than 150 cm, not first born, and growth faltering at six months of age) were associated independently with the odds of stunting at 36 months (p-values < 0.1). There were no interactions between them and gender (likelihood ratio p > 0.2). The most influential missing values occurred in our outcome, stunting at 36 months [see additional file 2]. However, we can conclude that the evidence for the associations we found is fairly robust to the missing values in our data.

**Table 4:** Crude odds of stunting at 36 months of age and (95% Confidence Intervals, CI) for the complete case analysis

		**COMPLETE CASE ANALYSIS**^1^**(N = 277)**		
**Risk factor**	**Number of children**	**Number (%) stunted**	**Crude OR (95% CI)**	**p-value**
Low birth weight				
No	245	136 (56%)	1	< 0.001
Yes	32	28 (88%)	5.61 (1.91-16.48)	
Beedi work in household				
No	150	75 (50%)	1	< 0.001
Yes	127	89 (70%)	2.34 (1.43-3.85)	
Maternal height				
>=150 cm	193	102 (53%)	1	< 0.001
< 150 cm	84	62 (74%)	2.51 (1.43-4.41)	
Growth faltering at 6 months, child either stunted, wasted or underweight				
No	153	77 (50%)	1	< 0.001
Yes	124	87 (70%)	2.32 (1.41-3.82)	
First born				
Yes	92	49 (53%)	1	0.157
No	185	115 (62%)	1.44 (0.87-2.39)	
SES class				
Class I (lower)	162	104 (64%)	1.64 (1.01-2.67)	
Class II (lower middle)	115	60 (52%)	1	0.045
Gender				
Male	138	86 (62%)	1	0.293
Female	139	78 (56%)	0.77 (0.48-1.25)	
Age at introduction of complementary food				
>= 4 months	177	111 (63%)	1	0.115
< 4 months	100	53 (53%)	0.67 (0.41-1.10)	
Maternal education				
None	78	53 (68%)	1	0.048
Primary and Middle	78	49 (63%)	0.80 (0.41-1.54)	
Higher and College	121	62 (51%)	0.50 (0.27-0.90)	
Maternal age				
<=23	165	97 (59%)	1	0.864
> 23	112	67 (60%)	1.04 (0.64-1.70)	
Days spent with major illness during first 3 years of life				
<=20 days	109	63 (58%)	1	0.203
21-41 days	89	48 (54%)	0.85 (0.49-1.50)	
> 41 days	79	53 (67%)	1.49 (0.81-2.72)	

^1 ^Only records which contained no missing values were included in the model; 42 missing values of the outcome stunting, 8 missing values of birth weight, 32 missing values of mother's height and 28 missing values of growth faltering at six months occurred in 96 children who were excluded from analysis.

**Table 5:** Multivariate odds of stunting at 36 months of age and (95% Confidence Intervals, CI) for the complete case analysis and the analysis with missing values imputed

	**COMPLETE CASE ANALYSIS**^1^**(N = 277)**		***IMPUTED ANALYSIS***^2^***(N = 373)***	
**Risk factor**	**Adjusted OR (95% CI)**	**p-value**	***Adjusted OR (95% CI)***	***p-value***

Low birth weight				
No	1	0.012	*1*	*0.010*
Yes	3.81 (1.20-12.1)		*3.63 (1.36-9.70)*	
Beedi work in household				
No	1	0.011	*1*	*0.031*
Yes	2.09 (1.18-3.70)		*1.74 (1.05-2.86)*	
Maternal height				
>=150 cm	1	0.042	*1*	*0.019*
< 150 cm	1.88 (1.01-3.47)		*2.02 (1.12-3.62)*	
Growth faltering at 6 months, child either stunted, wasted or underweight				
No	1	0.029	*1*	*0.032*
Yes	1.85 (1.06-3.22)		1.75 (1.05-2.93)	
First born				
Yes	1	0.069	*1*	*0.016*
No	1.77 (0.95-3.28)		*2.00 (1.14-3.51)*	
SES class				
Class I (lower)	1.60 (0.93-2.76)		*1.36 (0.82-2.26)*	
Class II (lower middle)	1	0.091	*1*	*0.237*
Gender				
Male	1	0.451	*1*	*0.244*
Female	0.81 (0.48-1.39)		*0.75 (0.46-1.22)*	
Age at introduction of complementary food				
>= 4 months	1	0.449	*1*	*0.353*
< 4 months	0.81 (0.46-1.40)		*0.79 (0.48-1.30)*	
Maternal education				
None	1	0.824	*1*	*0.596*
Primary and Middle	0.94 (0.45-1.97)		*0.77 (0.40-1.47)*	
Higher and College	0.81 (0.40-1.64)		*0.73 (0.39-1.36)*	
Maternal age				
<=23	1	0.640	*1*	*0.097*
> 23	0.87 (0.49-1.55)		*0.64 (0.38-1.08)*	
Days spent with major illness during first 3 years of life				
<=20 days	1	0.311	*1*	*0.103*
21-41 days	0.68 (0.36-1.29)		*0.62 (0.34-1.11)*	
> 41 days	1.14 (0.59-2.2)		*1.20 (0.66-2.20)*	

^1 ^Only records which contained no missing values were included in the model; 42 missing values of the outcome stunting, 8 missing values of birth weight, 32 missing values of mother's height and 28 missing values of growth faltering at six months occurred in 96 children who were excluded from analysis.

^2 ^Missing values imputed and adjusted analysis conducted with a random effects logistic regression, [see additional file 1].

Finally, when investigating risk factors for growth faltering at six months in 403 children with missing values imputed [see additional file 3], we found that low birth weight (OR: 4.71, 95% CI: 1.99-11.12) and maternal height less than 150 cm (OR:2.22, 95% CI: 1.25-3.95) were independently associated with the odds of growth faltering at 6 months. Illness was also associated with faltering at six months of age with the effect modified by gender (Wald p-value = 0.014). Boys with the highest days spent with any illness (> 6 weeks) during the first six months of life had 27.78 times (95% CI: 3.66-210.95) the odds of faltering compared with boys with the lowest illness (<=3 weeks) and girls with the highest days illness had 4.66 times (95% CI: 1.86-11.67) the odds of faltering compared with girls with the lowest illness.

## Discussion

Longitudinal studies are important to understand patterns of growth in children. We studied height and weight longitudinally in a birth cohort till three years of age in an urban slum in India. By age three years the majority of children (224/331, 68%) were stunted and/or wasted and/or underweight. Stunting was the predominant growth faltering, affecting 61% of children. Of those who were stunted, there were some children who were stunted only, but more than half were stunted and underweight only or stunted, underweight and wasted. We were able to present age-specific estimates of growth faltering due to the longitudinal nature of our study. In contrast, nationwide surveys report the percentage of children in cross-sectional studies who were stunted, wasted or underweight. The latest such survey in India, the National Family Health Survey-3 (NFHS-3) in 2005/6, estimated stunting among children under three at 25% [19] in Tamil Nadu (the state where our study was carried out), which is much lower than observed in our study. Unfortunately age-specific rates were not available for comparison.

Due to varied growth references and cut-offs used it can be difficult to compare studies directly. Furthermore little data on growth has been reported in Southern India. However, the rates of stunting, wasting and underweight we observed are broadly in line with other published studies of malnutrition among urban slum dwellers in northern India [11]. Although a common finding was that urban slum dwelling children had poorer growth than their rural or urban counterparts, NFHS-3 data sampled both urban and rural children and found little difference between them [19], although rates of growth among urban slum dwellers were not specified.

We found that growth faltering affected boys more than girls. At three years of age, 66% of boys and 56% of girls were stunted. Our findings are in contrast to other studies from India [11] and Pakistan [20] which showed greater levels of stunting in girls, possibly because they receive less preferential care than boys. Others in Bangladesh have found that gender differences may depend on the growth reference used [21]. In our setting, we found care was sought slightly more frequently for male children [13]. However we provided easy access to high quality monitoring and care for all children and this may have reduced the differences in stunting and the outcomes between the two genders.

Growth faltering has multi-factorial aetiology including poor nutrition and illness among other factors. Although morbidity was high it was primarily respiratory illness [[13], and Gladstone *et al.* unpublished]. More severe illnesses and mortality were low due to the close monitoring, support and free health care provided to the cohort. Compared with other developing country settings, the better health care received by our cohort could explain our finding that cumulative illness was not associated with stunting at three years of age. This suggests that nutrition might have played a greater role in the rates of stunting, wasting and underweight than illness. A total of 14 children received limited nutritional interventions of high calorie meals during the study and only after clinical diagnosis of Grade III malnutrition (51-60% of expected weight for age) of the Indian Academy of Pediatrics classification. Furthermore, we showed that the odds of stunting at 36 months were increased by growth faltering as early as six months of age. Independent of other effects, low birth weight was also a strong risk factor for growth faltering at 6 months and stunting at 36 months. All children started breastfeeding in this population although only a few continued to exclusively breastfed till six months of age (Rehman *et al.* unpublished). These findings suggest that in this setting the children are not getting the chance to recover from early growth faltering. It is possible that breastfeeding and/or weaning practices may have been inadequate and that maternal undernutrition, as suggested by the association we found between stunting at 36 months and maternal height, could contribute to the growth failures [22].

Wealth of the household could also impact on the rates of growth faltering that we observed. We did not find an association between stunting at 36 months and socio-economic status but this was because children in the study were a homogeneously low or low-middle socio-economic status. Poorer households in the area are involved with beedi work, which was associated with stunting at 36 months. In a previous publication we had also found that morbidity was associated with beedi work [13]. Finally, we found that firstborn children had lower odds of being stunted at 36 months. Smaller family size can mean there is less competition for food. In addition, mothers may have more time to care for their firstborn child.

Growth data was collected as a small part of the main rotavirus study, which after six months of age had very low rates of dropout. We obtained scheduled growth measurements on at least 88% of occasions. Thus, we are able to generalise our results within this population. There were some very low z-scores, in relation to the standard, for example less than -6SD, but these were cross-checked and most were found to be correct, hence we included children in our population who would be excluded from large-scale surveys where it is not possible to check individual children to the same degree. As growth was not the primary outcome of the study only single measurements were taken each month. We therefore have no measure of the reliability of the anthropometric measurements [23] so we may have misclassified some children. However, we *a priori *established that we would only exclude height measurements that were grossly out of line with a child's growth curve and that all weight measurements would be used as transcribed. The multiple imputations we carried out on the missing values and subsequent random effects logistic regression gave odds ratios and confidence intervals broadly in line with the complete case analysis. A sensitivity analysis that we carried out also showed our estimated odds ratios were fairly robust to the missing values in our data. This approach has given more confidence in the associations we identified.

We considered stunting, wasting and underweight together, to get an overall picture of the burden of malnutrition as suggested by Svedburg's "composite index of anthropometric failure (CIAF)" [24] which was modified to include a category for "underweight only" children. Nandy et al. [25] presented India-wide cross-sectional data for children from birth to 3 years using the NFHS-2 survey and used Svedburg's modified model to estimate overall malnutrition at 60%, a similar figure to the 68% we estimated at three years of age. However, they did not present age-specific nor gender-specific estimates and did not have longitudinal follow-up of children. Our study is able to expand upon their findings in presenting gender and age differences in the rates of stunting, wasting and underweight.

We found that growth faltering was common among these urban slum dwellers, even from one month of age. We looked at monthly transitions in growth and found that children with normal growth were highly likely to have normal growth in the next month. Similarly, children with a growth faltering were highly likely to have growth faltering the following month. Stunted children were more likely to stay stunted in the following month whereas wasted children were not as likely to stay wasted. This indicates the chronic nature of stunting; that once stunting occurs a child is more likely to remain stunted, while wasting could be corrected. The chronicity of stunting was especially so for children who were stunted in combination with underweight or underweight and wasted.

## Conclusion

Although complementary feeding, such as the Indian governmental 'mid-day meal' program, has been effective in decreasing severe malnutrition in school-going children, the early and chronic nature of the growth failures in pre-school children indicate that health policy makers should rethink strategies to address undernutrition, which is highly prevalent in countries such as India [11,26,27]. The finding that a child with growth faltering at six months is at greater risk of stunting at three years of age suggests that interventions such as improvements to complementary feeding and nutritional supplements as described by Bhutta et al [28] are urgently needed in such populations and should be targeted before and during pregnancy as well as during very early life.

## Competing interests

The authors declare that they have no competing interests.

## Authors' contributions

AMR carried out the statistical analysis and drafted the manuscript. BPG coordinated the study and assisted with statistical analysis and drafting the manuscript. VPV assisted with the analysis and interpretation of the data. JM assisted in the design and coordination of the study and with the statistical analysis. SJ assisted with the analysis of the data and critically revised the manuscript. GK conceived the study and assisted with the design and coordination of the study and helped to draft and revise the manuscript. All authors read and approved the final manuscript.

## Supplementary Material

Additional file 1 **Imputation of missing values**. Details are provided of covariates used in imputation models and imputed values. Click here for file

Additional file 2 **Sensitivity analyses for stunting at 36 months**. Details are provided of estimated odds ratios and confidence intervals for 16 sensitivity analysis models. Click here for file

Additional file 3 **Analysis of growth faltering at six months and sensitivity analyses**. Details are provided of estimated odds ratios and confidence intervals for our secondary outcome, growth faltering at six months, and for 8 sensitivity analysis models. Click here for file
