# Supplementary material for: Chronic growth faltering amongst a birth cohort of Indian children begins prior to weaning and is highly prevalent at three years of age
Source: Nutr J. 2009 Sep 29;8:44. doi: 10.1186/1475-2891-8-44 (PMC2761939; doi:10.1186/1475-2891-8-44)
Supplement: Additional file 1 — Imputation of missing values. Details are provided of covariates used in imputation models and imputed values. [file 1475-2891-8-44-S1.PDF]

Additional file 1: Imputation of missing values

Our primary outcome was the odds of stunting at our study endpoint, 36 months of age. Of the 452 children that began follow-up, 74 dropped out of the study and 5 died. At our study endpoint, 373 children were still under follow-up. We examined the associations between not completing follow-up and each of the potential risk factors (defined *a priori*) for our multivariate model and found associations with mother's education level and the age at introduction of complementary foods. We included these predictors of missingness in our multivariate model to give us an analysis that assumes our dropouts were so-called "missing at random" (MAR) [1].

In our "completers" cohort of 373 children we had missing values in our outcome variable and in three covariates (Table 1). Our complete case analysis excluded 96 (26%) children.

Table 1 – Variables in the multivariate logistic regression model for the odds of stunting at 36 months and numbers of missing and non-missing values

Variables	Number of missing values	Number of non-missing values
**Outcome**		
**Stunting at 36 months (stunted36)**	42	331
**Risk factors**		
**Low birth weight (lbw)**	8	365
**Beedi making in the household (beedi)**	0	373
**Mother's height (mheight)**	32	341
**Growth faltering at six months (growfalt6)**	28	345
**Not first born (firstborn)**	0	373
**SES Class (ses)**	0	373
**Sex (sex)**	0	373
**Age introduced complementary foods (compfood)**	0	373
**Mother's education attainment (medu)**	0	373
**Mother's age (mage)**	0	373
**Duration of major illness in the first three years of life (mjill)**	0	373

To use more of our data, we used standard multiple imputation methods to create 10 imputations of all missing values in our "completers" cohort [2]. We used all 452 children to create the imputed values. In addition to the list of variables in our multivariate model (shown in Table 1), we included (by default) the variables associated with missingness but also included other variables related to our missing variables as suggested by van Buuren *et al.* [3] (see Table 2 for list of variables used in each imputation model). We included the height-for-age z-score from 35 months of age in the imputation model for stunting at 36 months, which was not missing in 37/42 children with missing values of stunting at 36 months of age. We included the weight of the child from 1 month of age in the imputation model for low birth weight, which was not missing in 7/8 children with missing values for low birth weight. We included no addition variables in the imputation model for mother's height. Finally, we amended the core list of variables we included in the imputation model for growth faltering at six months to include illness in the first six months of life and its interaction with gender (as these were predictors in the multivariate model with

growth faltering at six months as the outcome) and also the height-for-age, weight-for-age and weight-for-height z-scores from 5 months of age which were not missing in any children with missing values for growth faltering at 6 months.

Table 2 – Details of multiple imputation models for the imputing of missing values

Variables with missing values	Model used in imputation	Variables used in imputation model
**Stunting at 36 months**	Logistic regression	lbw, beedi, mheight, growfalt6, firstborn, ses, sex, compfood, medu (two dummy variables), mage, mjill (two dummy variables), height-for-age z-score at 35 months
**Low birth weight**	Logistic regression	stunted36, beedi, mheight, growfalt6, firstborn, ses, sex, compfood, medu (two dummy variables), mage, mjill (two dummy variables), weight at 1 month of age
**Mother's height**	Logistic regression	stunted36, lbw, beedi, growfalt6, firstborn, ses, sex, compfood, medu (two dummy variables), mage, mjill (two dummy variables),
**Growth faltering at six months**	Logistic regression	stunted36, lbw, beedi, mheight, firstborn, ses, sex, compfood, medu (two dummy variables), mage, all illness in first six months of life (two dummy variables), interaction between sex and all illness (two dummy variables), height-for-age z-score at 5 months, weight-for-age z-score at 5 months, weight-for-height z-score at 5 months

The imputed values in our outcome, stunting at 36 months were more often "yes" the child was stunted, ranging from 25/42 to 33/42 missing values among the 10 imputation models (Table 3). Almost all missing values of low birth weight were imputed to be "no" the child did not have low birth weight, ranging from 7/8 to 8/8 (Table A.3). More often mother's height was imputed to be >=150cm than <150cm, ranging from 17/32 to 26/32 (Table 3). Growth faltering at six months was more evenly split with imputed values of "no" growth faltering ranging between 13/28 to 20/28 missing values (Table 3).

For our secondary outcome of growth faltering at six months, our "completers" cohort of 403 children still under follow-up had missing values in the outcome variable (32) and in two covariates, low birth weight (10) and mother's height (50). Our complete case analysis excluded 85 (21%) children. We investigated factors related to not completing follow-up to six months of age and found associations with duration of illness during the first six months (chi-square p-value<0.001) and the age at introduction of complementary foods (log-rank test p-value=0.0013). These predictors of missingness were included in our multivariate and imputation models by default. Distributions of imputed values were a similar pattern as for stunting at 36 months (Table 4).

Table 3 – Imputed values in each of 10 imputation models for the odds of stunting at 36 months

		IMPUTATION									
**Variable**	**Total missing values**	1	2	3	4	5	6	7	8	9	10
**Stunting at 36 months**	42										
**No**		9	11	12	11	15	11	14	10	12	17
**Yes**		33	31	30	31	27	31	28	32	30	25
**Low birth weight**	8										
**No**		8	8	7	8	7	7	8	8	7	7
**Yes**		0	0	1	0	1	1	0	0	1	1
**Mother's height**	32										
**>=150 cm**		23	18	23	21	17	18	23	26	25	18
**<150 cm**		9	14	9	11	15	14	9	6	7	14
**Growth faltering at six months**	28										
**No**		20	15	14	15	15	17	18	17	13	19
**Yes**		8	13	14	13	13	11	10	11	15	9

Table 4 – Imputed values in each of 10 imputation models for the odds of growth faltering at 6 months

		IMPUTATION									
**Variable**	**Total missing values**	1	2	3	4	5	6	7	8	9	10
**Growth faltering at six months**	32										
**No**		21	17	14	16	17	19	21	19	14	20
**Yes**		11	15	18	16	15	13	11	13	18	12
**Low birth weight**	10										
**No**		9	10	9	9	8	8	10	10	9	8
**Yes**		1	0	1	1	2	2	0	0	1	2
**Mother's height**	50										
**>=150 cm**		32	29	32	35	30	27	37	38	37	31
**<150 cm**		18	21	18	15	20	23	13	12	13	19

REFERENCES

1. Rubin DB: **Multiple imputation after 18+ years**. *Journal of the American Statistical Association* 1996, **91**(434):473-489.
2. Royston P: **Multiple imputation of missing values: update of ice**. *Stata Journal* 2005, **5**:527-536.
3. van Buuren S, Boshuizen HC, Knook DL: **Multiple imputation of missing blood pressure covariates in survival analysis**. *Stat Med* 1999, **18**(6):681-694.
