# Supplementary material for: Chronic growth faltering amongst a birth cohort of Indian children begins prior to weaning and is highly prevalent at three years of age
Source: Nutr J. 2009 Sep 29;8:44. doi: 10.1186/1475-2891-8-44 (PMC2761939; doi:10.1186/1475-2891-8-44)
Supplement: Additional file 2 — Sensitivity analyses for stunting at 36 months. Details are provided of estimated odds ratios and confidence intervals for 16 sensitivity analysis models. [file 1475-2891-8-44-S2.PDF]

Additional file 2: Sensitivity analyses for stunting at 36 months

To examine the stability of the estimated odds ratios we conducted a sensitivity analysis using our "completers" cohort of 373 children. All of our missing values occurred in four categorical variables each with two levels. We therefore in turn set the missing values alternatively to 0 and 1 in each of the four variables with missing values resulting in 2^4=16 models which could be considered the extremes of any imputation models (allocation for each model is shown in Table 1). Although this may not result in the very extremes of our estimated odds ratios it does allow us to examine which variable's missing values are most influential on our odds ratios [1].

The most influential variable was our outcome, stunting at 36 months (Table 2). When all the missing values of stunting were set to 0 (or "not stunted") there was no longer evidence of an association between stunting and maternal height nor stunting and growth faltering at six months. Evidence of an association between stunting and low birth weight, stunting and beedi making in the household and stunting and not being first born remained regardless of what values the missing values were given. Estimated ORs for other risk factors remained relatively stable also, regardless of the values missing values were given. Of the 42 children with missing stunting outcome, 37 children had their height-for-age z-score recorded at 35 months of age and 30/37 (81%) were stunted at 35 months of age. Given this further detail and knowing the high rate of stunting in observed data it seems much more likely that the missing values would tend to be that the child was stunted, rather than not. The lack of evidence for association with maternal height and growth faltering at six months occurred when missing values for stunting were not given realistic values. Our imputation models imputed the missing values to be more often "yes" stunted, and are more similar to the rates of stunting at 35 months (see additional file 1, Table 3).

Table 1 – Allocation of missing values for each of 16 sensitivity analyses

Variable	Model 1	Model 2	Model 3	Model 4	Model 5	Model 6	Model 7	Model 8	Model 9	Model 10	Model 11	Model 12	Model 13	Model 14	Model 15	Model 16
**Stunting at 36 months**																
**No**	x	X	x	x	x	x	x	x								
**Yes**									X	x	x	x	X	x	x	x
**Low birth weight**																
**No**	x		x	x			x		X		x	x			x	
**Yes**		x			x	x		x		x			X	x		X
**Mother's height**																
**>=150 cm**	x	x		x		x			X	x		x		x		
**<150 cm**			x		x		x	x			x		X		x	X
**Growth faltering at six months**																
**No**	x	x	x		x				X	x	x		X			
**Yes**				x		x	x	x				x		x	x	X

Table 2 – Estimated odds ratios (OR) and (95% CI) from 16 models with missing values set to 0 and 1 in turn

Risk factor	Model 1	Model 2	Model 3	Model 4	Model 5	Model 6	Model 7	Model 8
**Low birth weight**								
**No**	1	1	1	1	1	1	1	1
**Yes**	4.02 (1.71-9.44)	3.59 (1.66-7.75)	3.86 (1.65-9.03)	4.07 (1.74-9.56)	3.46 (1.60-7.49)	3.61 (1.67-7.81)	3.90 (1.67-9.13)	3.49 (1.61-7.54)
**Beedi work in household**								
**No**	1	1	1	1	1	1	1	1
**Yes**	1.62 (1.02-2.56)	1.64 (1.04-2.59)	1.63 (1.04-2.58)	1.60 (1.01-2.52)	1.65 (1.05-2.60)	1.61 (1.02-2.54)	1.61 (1.02-2.54)	1.62 (1.03-2.56)
**Maternal height**								
**>=150 cm**	1	1	1	1	1	1	1	1
**<150 cm**	1.46 (0.89-2.38)	1.39 (0.85-2.28)	1.46 (0.92-2.32)	1.47 (0.90-2.40)	1.40 (0.88-2.23)	1.41 (0.86-2.30)	1.48 (0.93-2.34)	1.42 (0.89-2.25)
**Growth faltering at 6 months, child either stunted, wasted or underweight**								
**No**	1	1	1	1	1	1	1	1
**Yes**	1.46 (0.92-2.31)	1.49 (0.94-2.35)	1.46 (0.92-2.31)	1.40 (0.89-2.19)	1.49 (0.94-2.35)	1.41 (0.90-2.21)	1.40 (0.89-2.19)	1.41 (0.90-2.21)
**First born**								
**Yes**	1	1	1	1	1	1	1	1
**No**	1.81 (1.08-3.01)	1.78 (1.07-2.96)	1.77 (1.06-2.96)	1.82 (1.09-3.03)	1.75 (1.05-2.91)	1.78 (1.07-2.96)	1.78 (1.07-2.97)	1.75 (1.05-2.92)
**SES class**								
**Class I (lower)**	1.18 (0.74-1.87)	1.16 (0.73-1.84)	1.17 (0.74-1.86)	1.16 (0.73-1.84)	1.16 (0.73-1.87)	1.14 (0.72-1.81)	1.16 (0.73-1.83)	1.14 (0.72-1.81)
**Class II (lower middle)**	1	1	1	1	1	1	1	1
**Gender**								
**Male**	1	1	1	1	1	1	1	1
**Female**	0.70 (0.45-1.09)	0.71 (0.46-1.10)	0.71 (0.46-1.09)	0.70 (0.45-1.09)	0.71 (0.46-1.10)	0.70 (0.45-1.09)	0.70 (0.45-1.09)	0.71 (0.46-1.09)
**Age at introduction of complementary food**								
**>= 4 months**	1	1	1	1	1	1	1	1
**<4 months**	0.86 (0.54-1.37)	0.86 (0.54-1.37)	0.88 (0.55-1.39)	0.85 (0.54-1.35)	0.88 (0.55-1.39)	0.85 (0.54-1.35)	0.87 (0.54-1.38)	0.86 (0.54-1.37)
**Maternal education**								
**None**	1	1	1	1	1	1	1	1
**Primary and Middle**	0.77 (0.43-1.38)	0.79 (0.44-1.41)	0.78 (0.43-1.39)	0.76 (0.42-1.36)	0.79 (0.44-1.42)	0.77 (0.43-1.38)	0.76 (0.43-1.37)	0.78 (0.43-1.40)
**Higher and College**	0.84 (0.52-1.33)	0.77 (0.43-1.38)	0.75 (0.42-1.33)	0.72 (0.40-1.29)	0.78 (0.44-1.40)	0.76 (0.42-1.35)	0.73 (0.41-1.31)	0.77 (0.43-1.37)
**Maternal age**								
**<=23**	1	1	1	1	1	1	1	1
**>23**	0.85 (0.52-1.33)	0.82 (0.51-1.31)	0.83 (0.52-1.33)	0.83 (0.52-1.32)	0.82 (0.51-1.31)	0.81 (0.51-1.30)	0.82 (0.51-1.32)	0.81 (0.51-1.30)
**Days spent with major illness during first 3 years of life**								
**<=20 days**	1	1	1	1	1	1	1	1
**21-41 days**	0.79 (0.47-1.34)	0.82 (0.48-1.39)	0.79 (0.46-1.33)	0.79 (0.47-1.34)	0.81 (0.48-1.38)	0.82 (0.48-1.39)	0.79 (0.46-1.33)	0.81 (0.48-1.38)
**>41 days**	1.08 (0.63-1.85)	1.10 (0.64-1.88)	1.07 (0.63-1.84)	1.11 (0.65-1.89)	1.09 (0.64-1.87)	1.12 (0.66-1.92)	1.10 (0.64-1.88)	1.12 (0.65-1.91)

Table 2 continued

Risk factor	Model 9	Model 10	Model 11	Model 12	Model 13	Model 14	Model 15	Model 16
**Low birth weight**								
**No**	1	1	1	1	1	1	1	1
**Yes**	3.17 (1.22-8.28)	3.05 (1.25-7.40)	2.89 (1.11-7.51)	3.11 (1.19-8.12)	2.79 (1.15-6.78)	2.98 (1.22-7.24)	2.82 (1.08-7.33)	2.71 (1.12-6.60)
**Beedi work in household**								
**No**	1	1	1	1	1	1	1	1
**Yes**	1.71 (1.05-2.79)	1.72 (1.06-2.80)	1.75 (1.07-2.85)	1.68 (1.03-2.75)	1.75 (1.07-2.86)	1.69 (1.04-2.76)	1.72 (1.05-2.82)	1.73 (1.05-2.82)
**Maternal height**								
**>=150 cm**	1	1	1	1	1	1	1	1
**<150 cm**	2.00 (1.15-3.48)	1.93 (1.11-3.36)	2.14 (1.28-3.58)	2.00 (1.15-3.48)	2.08 (1.24-3.48)	1.94 (1.11-3.38)	2.14 (1.28-3.58)	2.08 (1.24-3.49)
**Growth faltering at 6 months, child either stunted, wasted or underweight**								
**No**	1	1	1	1	1	1	1	1
**Yes**	1.77 (1.08-2.91)	1.79 (1.09-2.93)	1.77 (1.08-2.90)	1.88 (1.16-3.04)	1.78 (1.09-2.92)	1.89 (1.17-3.05)	1.87 (1.16-3.04)	1.88 (1.16-3.05)
**First born**								
**Yes**	1	1	1	1	1	1	1	1
**No**	1.94 (1.13-3.33)	1.93 (1.13-3.30)	1.86 (1.09-3.20)	2.00 (1.16-3.44)	1.85 (1.08-3.18)	1.98 (1.15-3.40)	1.92 (1.11-3.31)	1.90 (1.11-3.28)
**SES class**								
**Class I (lower)**	1.39 (0.85-2.26)	1.37 (0.84-2.24)	1.39 (0.85-2.26)	1.36 (0.83-2.21)	1.38 (0.84-2.24)	1.34 (0.82-2.19)	1.35 (0.83-2.21)	1.34 (0.82-2.19)
**Class II (lower middle)**	1	1	1	1	1	1	1	1
**Gender**								
**Male**	1	1	1	1	1	1	1	1
**Female**	0.73 (0.46-1.16)	0.73 (0.45-1.19)	0.73 (0.46-1.16)	0.73 (0.46-1.17)	0.73 (0.46-1.17)	0.73 (0.46-1.17)	0.73 (0.46-1.17)	0.73 (0.46-1.17)
**Age at introduction of complementary food**								
**>= 4 months**	1	1	1	1	1	1	1	1
**<4 months**	0.85 (0.52-1.38)	0.85 (0.52-1.39)	0.88 (0.54-1.44)	0.82 (0.50-1.35)	0.88 (0.54-1.45)	0.82 (0.50-1.35)	0.86 (0.52-1.40)	0.86 (0.52-1.41)
**Maternal education**								
**None**	1	1	1	1	1	1	1	1
**Primary and Middle**	0.81 (0.43-1.52)	0.83 (0.44-1.56)	0.82 (0.43-1.54)	0.78 (0.41-1.47)	0.84 (0.44-1.58)	0.79 (0.42-1.50)	0.78 (0.41-1.49)	0.80 (0.42-1.52)
**Higher and College**	0.72 (0.39-1.34)	0.75 (0.40-1.40)	0.74 (0.40-1.38)	0.70 (0.37-1.31)	0.77 (0.41-1.44)	0.73 (0.39-1.36)	0.72 (0.38-1.35)	0.74 (0.40-1.40)
**Maternal age**								
**<=23**	1	1	1	1	1	1	1	1
**>23**	0.68 (0.41-1.12)	0.67 (0.40-1.11)	0.68 (0.41-1.12)	0.66 (0.40-1.09)	0.67 (0.40-1.11)	0.65 (0.39-1.08)	0.65 (0.39-1.08)	0.65 (0.39-1.08)
**Days spent with major illness during first 3 years of life**								
**<=20 days**	1	1	1	1	1	1	1	1
**21-41 days**	0.63 (0.36-1.09)	0.64 (0.37-1.11)	0.62 (0.36-1.08)	0.63 (0.36-1.09)	0.63 (0.36-1.10)	0.64 (0.37-1.11)	0.62 (0.35-1.08)	0.63 (0.36-1.10)
**>41 days**	1.24 (0.69-2.23)	1.24 (0.69-2.25)	1.23 (0.68-2.21)	1.26 (0.70-2.28)	1.24 (0.69-2.24)	1.27 (0.70-2.29)	1.26 (0.70-2.27)	1.27 (0.70-2.29)

REFERENCES

1. **Guidelines for handling missing data in social science research**
[http://www.missingdata.org.uk/]
