# Supplementary material for: Chronic growth faltering amongst a birth cohort of Indian children begins prior to weaning and is highly prevalent at three years of age
Source: Nutr J. 2009 Sep 29;8:44. doi: 10.1186/1475-2891-8-44 (PMC2761939; doi:10.1186/1475-2891-8-44)
Supplement: Additional file 3 — Analysis of growth faltering at six months and sensitivity analyses. Details are provided of estimated odds ratios and confidence intervals for our secondary outcome, growth faltering at six months, and for 8 sensitivity analysis models. [file 1475-2891-8-44-S3.PDF]

Additional file 3: Analysis of growth faltering at six months and sensitivity analyses

By six months of age 403 (89%) children were still under follow-up. In a multivariate model investigating risk factors for growth faltering at six months (Table 1), we found two factors (low birth weight and maternal height less than 150cm) were associated independently with the odds of growth faltering at six months (p-values<0.01). Illness was also associated with faltering at six months of age with the effect modified by gender.

Table 1 – Complete case analysis and analysis with missing values imputed multivariate odds of growth faltering at 6 months of age and (95% CI)

	COMPLETE CASE ANALYSIS (N=318)		*IMPUTED ANALYSIS (N=403)*	
**Risk factor**	**Adjusted OR (95% CI)**	**p-value**	***Adjusted OR (95% CI)***	***p-value***
**Low birth weight**				
**No**	1	<0.001	*1*	*<0.001*
**Yes**	5.05 (2.02-12.58)		*4.71 (1.99-11.12)*	
**Maternal height**				
**>=150 cm**	1	<0.001	*1*	*0.007*
**<150 cm**	2.44 (1.44-4.14)		*2.22 (1.25-3.95)*	
**Boys**				
**Days spent with illness during the first 6 months of life**				
**<=20 days**	1		*1*	
**21-41 days**	3.30 (0.45-24.00)		*2.79 (0.39-20.00)*	
**>41 days**	21.52 (3.00-154.57)		*27.78 (3.66-210.95)*	
**Girls**		0.022		*0.014*
**Days spent with illness during the first 6 months of life**				
**<=20 days**	1		*1*	
**21-41 days**	1.87 (0.76-4.62)		*1.57 (0.65-3.80)*	
**>41 days**	4.01 (1.67-9.65)		*4.66 (1.86-11.67)*	
**Beedi work in household**				
**No**	1	0.422	*1*	*0.966*
**Yes**	0.81 (0.48-1.37)		*0.99 (0.59-1.66)*	
**First born**				
**Yes**	1	0.077	*1*	*0.163*
**No**	0.61 (0.35-1.06)		*0.67 (0.38-1.18)*	
**SES class**				
**Class I (lower)**	1.06 (0.63-1.76)		*0.92 (0.55-1.52)*	
**Class II (lower middle)**	1	0.833	*1*	*0.738*
**Age at introduction of complementary food**				
**>= 4 months**	1	0.605	*1*	*0.444*
**<4 months**	1.14 (0.69-1.91)		*1.21 (0.74-1.99)*	
**Maternal education**				
**None**	1	0.623	*1*	*0.644*
**Primary and Middle**	0.92 (0.48-1.78)		*0.95 (0.51-1.75)*	
**Higher and College**	0.74 (0.38-1.42)		*0.76 (0.41-1.43)*	
**Maternal age**				
**<=23**	1	0.090	*1*	*0.198*
**>23**	1.58 (0.93-2.70)		*1.42 (0.83-2.43)*	

To examine the stability of the estimated odds ratios we conducted a sensitivity analysis using our "completers" cohort of 403 children. All of our missing values occurred in three categorical variables each with two levels. We therefore in turn set the missing values alternatively to 0 and 1 in each of the three variables with missing values resulting in 2^3=8 models which could be considered the extremes of any imputation models (allocation for each model is shown in Table 2). Although this may not result in the very extremes of our estimated odds ratios it does allow us to examine which variable's missing values are most influential on our odds ratios [1].

Table 2 – Allocation of missing values for each of 8 sensitivity analyses

Variable	Model 1	Model 2	Model 3	Model 4	Model 5	Model 6	Model 7	Model 8
**Growth faltering at six months**								
**No**	x	x	x	x				
**Yes**					X	x	x	x
**Low birth weight**								
**No**	x		x		X		x	
**Yes**		x		x		x		x
**Mother's height**								
**>=150 cm**	x	x			X	x		
**<150 cm**			x	x			x	x

The most influential variable was our outcome, growth faltering at six months (Table 3). When all the missing values of growth faltering were set to 0 (or "no" growth faltering) estimated ORs moved closer to 1. When all the missing values of growth faltering were set to 1 (or "yes" growth faltering) there was weak evidence of an association between growth faltering and being first born, and growth faltering and having an older mother where no evidence of association was seen before. The two main effects we saw in our complete case analysis and imputed analysis remained regardless of what values the missing values were given, and in all but one case the estimated ORs were closer to 1. The interaction between gender and days spent with illness remained strong and the ORs were similar to our other analyses (in Table 1). Of the 31 children with missing values for growth faltering at six months, 17/31 had a growth faltering at five months of age which is a similar allocation to our imputation models (see additional file 1, Table 4).

Table 3 – Estimated odds ratios (OR) and (95% CI) from 8 models with missing values set to 0 and 1 in turn

Risk factor	Model 1	Model 2	Model 3	Model 4	Model 5	Model 6	Model 7	Model 8
**Low birth weight**								
**No**	1	1	1	1	1	1	1	1
**Yes**	3.83 (1.86-7.88)	2.91 (1.54-5.49)	3.62 (1.77-7.42)	2.76 (1.46-5.20)	3.90 (1.80-8.47)	2.95 (1.51-5.76)	3.69 (1.70-7.99)	2.79 (1.43-5.46)
**Maternal height**								
**>=150 cm**	1	1	1	1	1	1	1	1
**<150 cm**	2.24 (1.39-3.60)	2.16 (1.34-3.47)	1.90 (1.23-2.95)	1.83 (1.18-2.84)	2.13 (1.32-3.45)	2.06 (1.27-3.33)	1.86 (1.20-2.88)	1.79 (1.16-2.77)
**Boys: Days spent with illness during the first 6 months of life**								
**<=20 days**	1	1	1	1	1	1	1	1
**21-41 days**	3.03 (0.52-17.69)	2.89 (0.49-16.86)	3.10 (0.54-17.90)	2.96 (0.51-17.03)	4.66 (0.83-25.99)	4.44 (0.80-24.72)	4.71 (0.85-26.07)	4.50 (0.82-24.80)
**>41 days**	22.87 (4.02-129.95)	25.13 (4.45-142.06)	21.19 (3.76-119.37)	23.23 (4.15-130.06)	29.05 (5.22-161.61)	31.50 (5.69-174.48)	26.99 (4.89-148.88)	29.26 (5.33-160.73)
**Girls: Days spent with illness during the first 6 months of life**								
**<=20 days**	1	1	1	1	1	1	1	1
**21-41 days**	1.76 (0.79-3.94)	1.71 (0.76-3.83)	1.78 (0.80-3.97)	1.73 (0.78-3.85)	1.80 (0.82-3.95)	1.75 (0.80-3.85)	1.82 (0.83-3.98)	1.77 (0.81-3.86)
**>41 days**	4.32 (1.98-9.44)	4.45 (2.04-9.70)	4.15 (1.91-9.03)	4.27 (1.97-9.25)	4.51 (2.08-9.80)	4.62 (2.13-10.02)	4.35 (2.01-9.42)	4.47 (2.07-9.64)
**Beedi work in household**								
**No**	1	1	1	1	1	1	1	1
**Yes**	0.94 (0.59-1.48)	0.98 (0.62-1.53)	0.99 (0.63-1.55)	1.03 (0.66-1.61)	1.02 (0.65-1.60)	1.06 (0.68-1.66)	1.07 (0.68-1.67)	1.11 (0.71-1.73)
**First born**								
**Yes**	1	1	1	1	1	1	1	1
**No**	0.72 (0.44-1.18)	0.69 (0.42-1.13)	0.70 (0.43-1.15)	0.67 (0.41-1.09)	0.65 (0.40-1.06)	0.62 (0.38-1.01)	0.63 (0.39-1.03)	0.61 (0.37-0.99)
**SES class**								
**Class I (lower)**	0.81 (0.51-1.29)	0.80 (0.51-1.27)	0.81 (0.52-1.29)	0.80 (0.51-1.27)	1.05 (0.67-1.66)	1.04 (0.66-1.63)	1.06 (0.67-1.66)	1.04 (0.67-1.63)
**Class II (lower middle)**	1	1	1	1	1	1	1	1
**Age at introduction of complementary food**								
**>= 4 months**	1	1	1	1	1	1	1	1
**<4 months**	1.09 (0.69-1.72)	1.09 (0.69-1.72)	1.13 (0.71-1.78)	1.12 (0.71-1.77)	1.31 (0.84-2.06)	1.31 (0.83-2.05)	1.36 (0.86-2.13)	1.35 (0.86-2.11)
**Maternal education**								
**None**	1	1	1	1	1	1	1	1
**Primary and Middle**	0.88 (0.50-1.56)	0.91 (0.51-1.61)	0.93 (0.53-1.64)	0.95 (0.54-1.68)	1.05 (0.59-1.85)	1.07 (0.61-1.89)	1.09 (0.62-1.92)	1.12 (0.64-1.97)
**Higher and College**	0.77 (0.44-1.37)	0.81 (0.46-1.44)	0.80 (0.45-1.42)	0.84 (0.48-1.49)	0.86 (0.49-1.50)	0.90 (0.52-1.58)	0.89 (0.51-1.57)	0.93 (0.53-1.64)
**Maternal age**								
**<=23**	1	1	1	1	1	1	1	1
**>23**	1.36 (0.86-2.17)	1.34 (0.85-2.14)	1.39 (0.88-2.22)	1.38 (0.87-2.18)	1.57 (0.99-2.48)	1.54 (0.98-2.44)	1.59 (1.01-2.52)	1.57 (0.99-2.48)

REFERENCES

1. **Guidelines for handling missing data in social science research** [http://www.missingdata.org.uk/]
